# IL-7 in autoimmune diseases: mechanisms and therapeutic potential

**DOI:** 10.3389/fimmu.2025.1545760

**Published:** 2025-04-17

**Authors:** Ziyuan Zeng, Hanxiao Mao, Qirong Lei, Yuanmin He

**Affiliations:** Department of Dermatology, The Affiliated Hospital of Southwest Medical University, Luzhou, Sichuan, China

**Keywords:** IL-7, autoimmune diseases, cytokine, T cells, B cells, therapeutic targets

## Abstract

Interleukin-7 (IL-7) is a pleiotropic cytokine that plays a crucial role in the development, homeostasis, and function of the immune system. Growing evidence has demonstrated that IL-7 is involved in the pathogenesis of various autoimmune diseases including rheumatoid arthritis (RA), systemic lupus erythematosus (SLE), type 1 diabetes (T1D) and multiple sclerosis (MS). This review aims to summarize the current understanding of the role of IL-7 in autoimmune diseases, focusing on its mechanisms of action, implications for disease progression, and potential therapeutic applications. Produced by stromal cells, IL-7 binds to IL-7 receptor (IL-7R) on diverse immune cells. It is crucial for T cell development, survival, and proliferation. In autoimmune diseases, it activates and expands autoreactive T cells and influences B cell function, potentially leading to autoantibody production. The review further delves into the role of IL-7 in different autoimmune diseases. In RA, elevated IL-7/IL-7R promotes memory T cell survival, cytokine production, and influences B cells and monocytes to contribute to inflammation and joint damage. In SLE, elevated soluble form of IL-7R is associated with disease activity, promoting the survival of autoreactive T cells and enhancing the production of pro-inflammatory cytokines. In MS, genetic variations in the IL-7R gene are linked to disease susceptibility, and IL-7 impacts the survival and differentiation of T cell subsets involved in multiple sclerosis pathogenesis. For T1D, IL-7 affects the function of immune cells that attack pancreatic β cells. Given its central role in autoimmune processes, targeting the IL-7/IL-7R axis holds great therapeutic potential. By modulating IL-7 signaling, it may be possible to restore immune tolerance, reduce the activation of autoreactive immune cells, and alleviate disease symptoms. Understanding the complex mechanisms of IL-7 in autoimmune diseases is essential for the development of effective and targeted therapies.

## 1 Introduction

Autoimmune diseases are a cluster of disorders characterized by pathological autoimmune reactions within the body, triggered by various factors. These abnormal immune responses involve inflammatory processes that damage and injure the body’s own cellular components, leading to tissue destruction and organ dysfunction (1). Autoimmune diseases can be recurrent and chronic, usually involving the patient ‘s bones, muscles, joints and surrounding tissues (2). Autoimmune diseases are traditionally classified into organ-specific (where the immune response is directed against a particular tissue or organ) and systemic autoimmune diseases (where the immune response affects multiple organs). Systemic autoimmune diseases encompass conditions such as systemic lupus erythematosus (SLE), rheumatoid arthritis (RA), and Sjogren’s syndrome (pSS), where the immune response impacts various organ systems. Organ-specific autoimmune diseases include type 1 diabetes mellitus (targeting pancreatic beta cells), multiple sclerosis (affecting the central nervous system), and Hashimoto’s thyroiditis (targeting the thyroid gland) (1). Prevalence data collated from multiple surveys of various autoimmune diseases in many parts of the world confirm the overall upward trend. It is estimated that the total incidence and prevalence of autoimmune diseases worldwide are increasing by 19.1% and 12.5% per year, respectively (3). In fact, autoimmune diseases affect approximately 10% of the world’s population, causing great suffering to patients, and are also a major global socio-economic problem (4, 5).

As an important member of the cytokine family, interleukin-7 (IL-7) has received extensive attention in the research field of autoimmune diseases in recent years. Different from other common pro-inflammatory or anti-inflammatory cytokines, IL-7 has pleiotropism and unique biological functions in the immune system. It was initially found to play an indispensable role in the development, survival, and proliferation of T lymphocytes and plays a key role in maintaining T cell homeostasis (6–8). However, an increasing number of studies have shown that IL-7 also plays a complex and important role in the pathological process of autoimmune diseases (9). The mechanism of action of IL-7 in autoimmune diseases is complex and diverse, which involves multiple immune cell subsets and signaling pathways. IL-7 may play a role in the progression of autoimmune diseases by promoting the expansion of self-reactive clones (10). In addition, targeted therapy of the IL-7/IL-7Rα pathway has shown therapeutic effects in multiple autoimmune mouse models, indicating the important role of IL-7 in autoimmune inflammation (11). Genetic variations in the IL-7 receptor (IL-7R) are also associated with the susceptibility to autoimmune diseases. Study (12) has shown that polymorphisms in the IL7RA gene are associated with the susceptibility to diseases such as type 1 diabetes. These polymorphisms may regulate the biological activity of IL-7 by affecting the expression of soluble and membrane-bound IL-7Rα. In some cases, polymorphisms in IL-7R may promote the occurrence of autoimmune diseases by affecting the availability and biological activity of IL-7 (13). As a result, IL-7 becomes a highly promising target for studying the pathogenesis and treatment strategies of autoimmune diseases.

**Figure 1:**
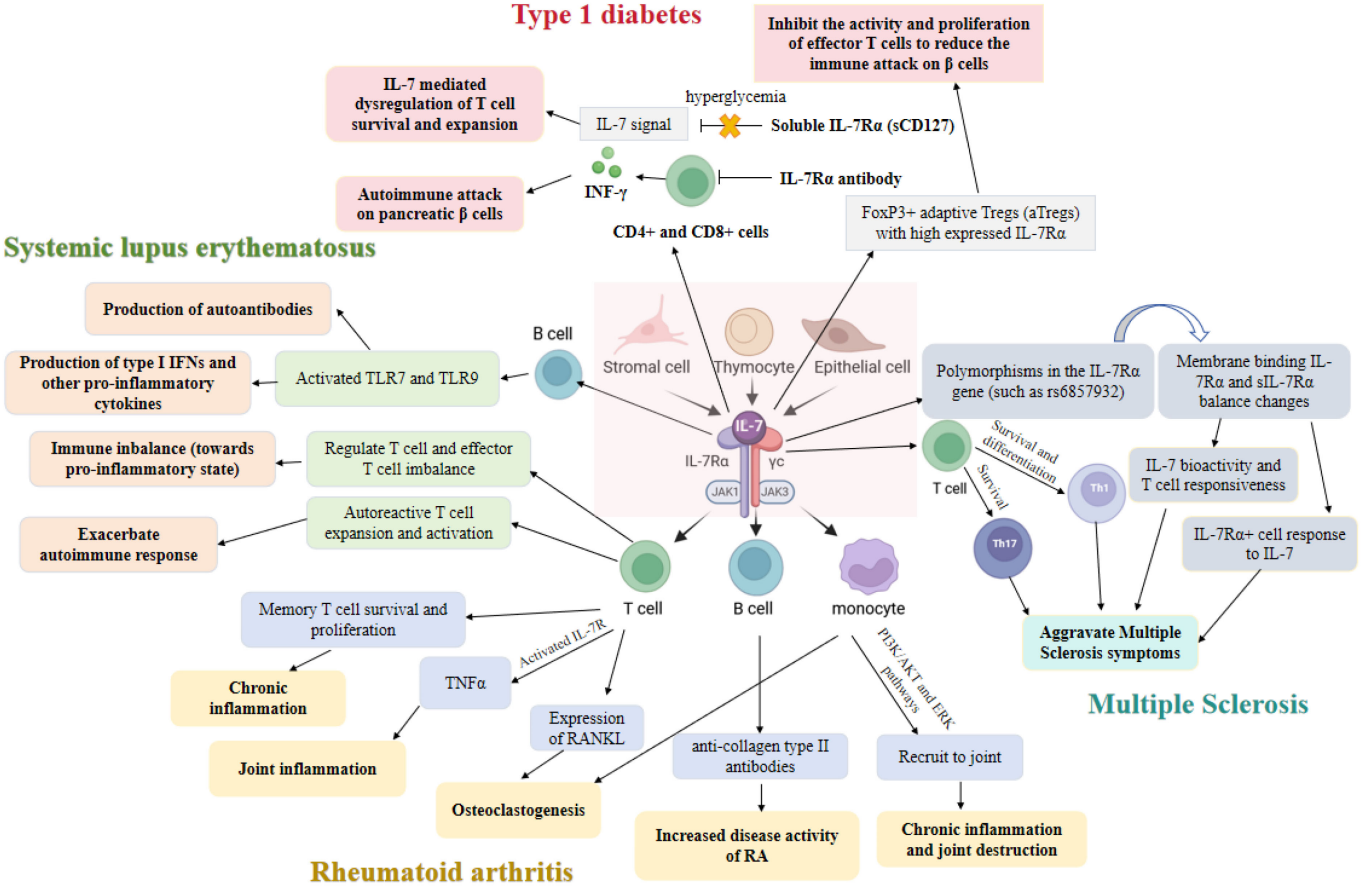
The role of interleukin-7 (IL-7) and its receptors in autoimmune diseases.

In view of the increasing importance of IL-7 in autoimmune diseases, and the current understanding of its mechanism of action and therapeutic potential is still to be systematically integrated and further explored, our review aimed to comprehensively review the research progress of IL-7 in autoimmune diseases. Firstly, we elaborated on the biological characteristics of IL-7 and its role in normal immune regulation. Next, the expression changes of IL-7 in different types of autoimmune diseases and the specific mechanisms involved in the occurrence and development of the disease were discussed (**Figure 1**). Finally, we summarized the current research status of treatment strategies targeting IL-7, including the progress of clinical trials and the challenges faced.

## 2 IL-7 and its receptor

IL-7 is a pleiotropic cytokine that plays a crucial role in the development, survival, and function of lymphoid cells. As a type I cytokine with a four α-helical bundle structure typical of the hematopoietin family, IL-7 is encoded by a single gene located on chromosome 8q12-13 in humans and chromosome 11 in mice (14). The mature form of IL-7 consists of 155 amino acids and has a molecular weight of approximately 25 kDa (6). IL-7 is secreted as a monomer and functions as both a growth factor and a survival factor for various immune cells. It is primarily produced by stromal cells in the bone marrow and thymus but can also be synthesized by epithelial cells, fibroblasts, and keratinocytes (15, 16). IL-7 exerts its effects through binding to IL-7R, a heterodimeric receptor composed of the IL-7Rα (CD127) and common gamma chain (γc, or IL-2Rγ also known as CD132) (6). IL-7Rα subunit binds IL-7 with high affinity, while γc subunit does not bind IL-7 directly but enhances the binding affinity and stability of the complex. The γc subunit is shared among several cytokine receptors, including those for IL-2, IL-4, IL-9, IL-15, and IL-21, indicating its critical role in immune regulation (17–19). This interaction triggers intracellular signaling pathways that regulate gene expression, cell proliferation, and differentiation. Binding of IL-7 to its receptor initiates a cascade of intracellular signaling events. The primary pathway involves the activation of Janus kinases (JAKs), particularly JAK1 and JAK3, which are associated with the IL-7Rα and γc subunits, respectively (20, 21). Activation of these kinases leads to the phosphorylation of signal transducer and activator of transcription (STAT) proteins, predominantly STAT5. Phosphorylated STAT5 proteins form dimers that translocate to the nucleus and regulate the transcription of target genes involved in cell proliferation, differentiation, and apoptosis inhibition (22). Additionally, IL-7 signaling can activate other pathways such as PI3K/AKT and MAPK/ERK, which contribute to cell survival and metabolic reprogramming (23).

### 2.1 IL-7/IL-7R in T cells

The IL-7 and its receptor play pivotal roles in the development, differentiation, and maintenance of T cells (24, 25), which are essential components of the adaptive immune system. The IL-7/IL-7R signaling axis is indispensable for the early stages of T cell development, particularly in the thymus, where it supports the survival of double-negative (DN) thymocytes, which lack both CD4 and CD8 surface markers (26–28). These DN thymocytes can be further categorized into four subsets (DN1-DN4), with IL-7 signaling being critical for the transition from DN2 to DN3 stages (27). This process involves the activation of the JAK and STAT pathways, particularly JAK1, JAK3, and STAT5, which promote the expression of anti-apoptotic proteins like Bcl-2, thereby ensuring cell survival (27). In addition to its role in early T cell development, IL-7 is also essential for the maintenance of mature T cells in the periphery. IL-7R expression is tightly regulated and is crucial for the homeostasis of naive and memory T cells (28–30). The IL-7R expression is modulated by various factors, including glucocorticoids and tumor necrosis factor-alpha (TNF-α) (29). In the periphery, IL-7 signaling supports the survival of naive T cells by maintaining their metabolic activity and preventing atrophy (30). This is achieved through the regulation of glycolysis and the maintenance of mitochondrial function, which are essential for T cell viability (30). IL-7 also plays a critical role in the homeostatic proliferation of T cells, particularly in lymphopenic conditions where IL-7 levels are elevated (28). This homeostatic proliferation is essential for the restoration of T cell numbers following depletion due to infections or other causes. Interestingly, IL-7 signaling must be intermittent rather than continuous to prevent cytokine-induced cell death (CICD) in naive CD8+ T cells (27). Continuous IL-7 signaling can lead to excessive proliferation, production of interferon-gamma (IFN-γ), and subsequent cell death, highlighting the importance of regulated IL-7 signaling for T cell homeostasis (27, 31). The IL-7/IL-7R axis is also involved in the differentiation of T cell subsets. For instance, IL-7 signaling through the IL-7Rα chain is necessary for the generation of early T lineage progenitors (ETPs) in the thymus, which are the most immature thymocyte population (32). ETPs require IL-7 for their proliferation and survival, and defects in IL-7 or IL-7R can lead to a marked reduction in ETP numbers (32). Furthermore, IL-7 signaling activates both the phosphatidylinositol 3-kinase (PI3K) and STAT5 pathways, which have distinct roles in T cell development. While PI3K is essential for the survival and proliferation of T cell precursors, STAT5 is more involved in their differentiation (33, 34). The balance between these pathways is crucial for proper T cell development and homeostasis, as demonstrated by studies showing that mutations affecting these pathways can lead to impaired T cell development and altered immune responses (34).

### 2.2 IL-7/IL-7R in B cells

The IL-7/IL-7R signaling axis is crucial for various stages of B cell development, from early progenitors to mature B cells (24). IL-7 is indispensable for the commitment of progenitor cells to the B cell lineage. It works in concert with transcription factors such as E2A, EBF, and Pax-5 to regulate B cell commitment and Ig rearrangement by modulating FoxO protein activation and Rag enhancer activity (35). The IL-7R signaling is tightly regulated by suppressor of cytokine signaling (SOCS) proteins, which ensure that appropriate IL-7 signals are transmitted for efficient B cell development (35). In murine models, IL-7 is critical for B lymphopoiesis, influencing the differentiation of IL-7-responsive cells *in vitro*. The differentiation process is modulated by CD45, pre-B cell receptor (pre-BCR), and other downstream signaling molecules (36). The IL-7R signaling is necessary for the transition from pre-pro-B cell stage to further maturational stages by up-regulating the expression of the B cell-specific transcription factor EBF and its target genes (37). This stage transition is crucial for the formation of a transcription factor network that determines B lineage specification (37). The interplay between IL-7R and pre-BCR signaling is essential for the differentiation of B cell precursors. Pre-BCR-deficient pro-B cells exhibit a failure to proliferate in response to IL-7, which can be overcome with increasing amounts of IL-7. This modulation of the IL-7 dose-response threshold is dependent on pre-BCR expression, allowing for selective expansion of pre-BCR+ cells under conditions where IL-7 is limiting (38). This fine-tuning of IL-7 responses ensures positive selection of pre-BCR+ pro-B cells and limits the duration of IL-7 responsiveness, leading to differentiation into an IL-7-unresponsive pre-B cell stage (38). IL-7 also plays a role in the suppression of premature Ig light-chain rearrangement in human B-cell development. CD127+ cells, which express the IL-7Rα chain, show differential Ig gene rearrangement compared to CD127- cells. Neutralization of IL-7 in cultures leads to an increase in Ig light-chain rearrangements in CD127+ cells, highlighting IL-7’s role in regulating Ig gene rearrangement (39). IL-7 and IL-7R are required for the development and maintenance of innate lymphoid cells (ILCs), which are essential for lymphoid structure generation and barrier defense. Deregulation of IL-7/IL-7R signaling can promote cancer development, making it a potential target for therapeutic interventions (26). In the context of leukemia, PELI2, an E3 ubiquitin ligase, regulates early B-cell progenitor differentiation via IL-7R expression. PELI2 interacts with and stabilizes PU.1 and TCF3 proteins through K63-polyubiquitination, which is necessary for IL-7R expression. The deletion of PELI2 leads to defects in B cell development, which can be restored by overexpression of PU.1. This underscores the significance of PELI2 in both normal B lymphopoiesis and malignant B-cell acute lymphoblastic leukemia (BCP-ALL) (40).

### 2.3 IL-7/IL-7R in NK cells

NK cells are critical components of the innate immune system, responsible for early defense against infections and tumor surveillance. The IL-7/IL-7R signaling pathway has been extensively studied for its effects on lymphoid cells, but its specific role in NK cell biology is complex and multifaceted. One of the key roles of IL-7 in NK cell biology is enhancing the survival of specific NK cell subsets. Research (41) has shown that IL-7Rα is specifically expressed in the CD56^bright^ noncytotoxic cytokine-producing NK subset. This expression is thymus-independent, which is a notable difference from what is observed in mice. IL-7 promotes the survival of the CD56^bright^ NK subset by inhibiting apoptosis and increasing the expression of the anti-apoptotic protein BCL2. However, IL-7 does not increase NK cell cytotoxicity, interferon-γ production, or the expression of activation markers, indicating that IL-7 and IL-15 play different roles in NK cell homeostasis and activation. Interestingly, the role of IL-7 in NK cell development appears to be context-dependent. In HCV and HIV infections, IL-7-induced NK cell functions are inhibited, manifested as reduced cytotoxic activity and impaired STAT5 signaling (42). In IL-7R-deficient mice (43), NK cell development is not entirely abrogated, suggesting that other cytokines can compensate for the lack of IL-7 signaling. This is in contrast to the development of γδ T cells, which is completely blocked in the absence of IL-7R signaling, highlighting the unique requirements of different immune cell lineages for IL-7. This finding underscores the complexity of cytokine signaling in immune cell development and the potential for redundancy among different cytokines. Moreover, IL-7’s role extends beyond the development of NK cells to their maintenance in specific tissues. For example, hepatocyte-derived IL-7 is crucial for the maintenance of intrahepatic NK cells. Conditional knockout study (44) in mice has shown that the absence of IL-7 in hepatocytes leads to a significant reduction in the number of NKT and T cells in the liver, although NK cell numbers remain unchanged. This suggests that while IL-7 is vital for certain liver-resident lymphocytes, NK cells in the liver may rely on additional or alternative sources of cytokines for their maintenance.

### 2.4 IL-7/IL-7R in ILCs

The role of IL-7 in innate lymphocytes is multifaceted and involves their development, function, and maintenance. IL-7 regulates the fate and function of ILCs by binding to IL-7R, affecting its role in immune defense, tissue repair, and disease (45). IL-7 plays a key role in the development of ILCs. ILCs have similar characteristics to T cell subpopulations, but lack specific antigen receptors. IL-7 and thymic stromal lymphopoietin (TSLP) affect the development and function of ILCs by binding to IL-7Rα (46). In particular, IL-7 signaling is essential for the development of ILC2, the second class of innate lymphocytes. Studies have shown that loss of IL-7 signaling leads to a decrease in the number of ILC2 and the expression of GATA3, while overexpression of IL-7 leads to amplification of ILC2 and an increase in GATA3 expression (47). IL-7 not only plays a role in the development of ILCs, but also plays an important role in their function. IL-7 signaling is particularly important for the regulation of ILC2 function in the lungs. The study found that loss of IL-7 signaling led to impaired production of IL-5, IL-13, and amphiregulin in lung ILC2 following influenza virus infection (48). In addition, IL-7 regulates the function of ILC2 by enhancing the expression of GATA3 and CD25 (48). IL-7 also plays a key role in the maintenance of ILCs. The control of ILCs by IL-7 at different anatomical locations is a complex issue because IL-7 receptors are expressed not only on ILCs, but also on surrounding neighboring cells such as vascular endothelial cells and mesenchymal cells, which compete with ILCs for limited amounts of IL-7 (49). In the lungs, IL-7 is expressed by bronchoalveolar epithelial cells and lymphoendothelial cells (LECs) and controls the activation and maintenance of lung ILC2 to varying degrees in airway inflammation (50).

### 2.5 IL-7/IL-7R in macrophages

IL-7 and IL-7R play a pivotal role in the development, differentiation, and maintenance of macrophages, particularly tissue-resident macrophages (TRMs). These macrophages are essential for tissue homeostasis and immune responses. The IL-7/IL-7R signaling axis is crucial during fetal development, influencing the establishment and survival of macrophage populations in various tissues. Research has demonstrated that IL-7R signaling is integral to the development of fetal macrophages. For instance, IL-7R deletion or pharmacological blockade during fetal development significantly impairs the establishment of macrophage populations in the liver, lung, brain, and epidermis. This impairment is due to reduced survival and proliferation of macrophages, as evidenced by increased apoptosis and decreased proliferation in these tissues (51). Further studies have shown that IL-7R signaling enhances the survival of fetal macrophages by preventing apoptosis, particularly in the liver and lung, where IL-7R blockade leads to a marked increase in apoptotic cells (52).The dynamic regulation of IL-7R expression is another critical aspect of macrophage development. During fetal development, IL-7R expression is upregulated as monocytes differentiate into macrophages. This upregulation is essential for the transition from monocytes to macrophages, indicating that IL-7R plays a direct role in macrophage differentiation (53). Additionally, *in vitro* studies have confirmed that fetal monocytes can differentiate into IL-7R+ macrophages, further supporting the importance of IL-7R in macrophage development (53). In esophageal squamous cell carcinoma (ESCC), the interaction between cancer cells and macrophages induces IL-7R expression in cancer cells. This overexpression promotes cancer cell survival, growth, and migration through the activation of the Akt and Erk1/2 signaling pathways. High IL-7R expression in ESCC is associated with poor prognosis, highlighting the potential of IL-7R as a therapeutic target in cancer (54). Moreover, Synovial macrophages in RA exhibit elevated IL-7R expression, which is associated with increased osteoclastogenesis and joint inflammation. IL-7-driven arthritis is characterized by an increase in IL-7R+ macrophages, which contribute to disease severity by promoting the differentiation of inflammatory M1 macrophages and bone-eroding osteoclasts. Anti-TNF therapy, which reduces IL-7R expression in macrophages, has been shown to ameliorate IL-7-induced arthritis, further underscoring the role of IL-7R in inflammatory diseases (55).

### 2.6 IL-7/IL-7R in non-classical T cells (NKT and γδT cells)

IL-7 plays a key role in the survival and homeostasis of NKT cells. Studies have shown that IL-17-produced NKT cells (NKT17 cells) have different survival needs than other NKT cells. Unlike conventional NKT cells, which rely on IL-15 for maintenance, RORγt+ NKT cells are completely dependent on IL-7. IL-7 promotes the expansion of NKT17 cells in a T cell receptor-independent manner, thereby supporting homeostasis (56). In addition, IL-7 regulates the survival of NKT17 cells through the PI3K/AKT/mTOR signaling pathway, rather than by enhancing STAT protein signaling. This effect of IL-7 has also been observed in other cell types, such as ROR gamma t+ innate lymphocytes and CD4+ Th17 cells, suggesting that these functionally similar cells share common survival needs (56). Further research showed that the survival of NKT cells does not depend on IL-15, but on IL-7. In IL-15 deficient mice, the number of peripheral NKT cells remained intact, while IL-7 deficiency significantly reduced the number of NKT cells. IL-7 promotes NKT cell survival and homeostasis through downstream STAT5 signaling (57).

IL-7 is essential for the development of γδT cells. Studies have shown that in mice lacking IL-7 receptor alpha, γδT cells are completely absent, while natural killer cells (NK cells) develop and function normally (58). This suggests that the function of IL-7Rα is necessary for the development of γδT cells. In IL-7 deficient mice, the development of γδT cells was severely inhibited, especially in the skin, intestine, liver, and spleen (43). In addition, IL-7 deficiency leads to impaired maturation of fetal γδT cells (59). IL-7 also plays an important role in the development of γδT cells in the lungs. The study found that IL-7 was able to rescue gamma-delta T cell precursors in the lung epithelium *in vitro* and *in vivo* in mice lacking thymus (60). This suggests that IL-7 plays an important regulatory role in the development and maintenance of γδT cells.

In conclusion, IL-7 plays a key role in the survival and development of NKT cells and gamma-delta T cells. For NKT cells, IL-7 supports survival and homeostasis through unconventional signaling pathways, while for γδT cells; IL-7 is integral to their development.

## 3 IL-7 in autoimmune diseases

### 3.1 Rheumatoid arthritis

Elevated levels of IL-7 and IL-7R have been observed in the synovial fluid and tissues of RA patients, correlating with increased disease activity (61, 62). This suggests that IL-7/IL-7R signaling is a critical driver of the inflammatory processes in RA. One of the primary roles of IL-7 in RA is its effect on T cells. IL-7 promotes the survival and proliferation of memory T cells, which are crucial for sustaining chronic inflammation (63). The activation of IL-7R on T cells leads to the production of pro-inflammatory cytokines, such as TNF-α, which further exacerbate the inflammatory milieu in the joints (61, 64). Additionally, IL-7Rα expression is significantly higher on CD4+ T cells in RA synovial fluid and tissue compared to peripheral blood, indicating a localized activation of these cells in the inflamed joints (62). IL-7 also influences other immune cells involved in RA. For instance, IL-7R+ B cells have been shown to act pro-inflammatory in collagen-induced arthritis (CIA), a mouse model of RA. These B cells contribute to the disease by increasing the production of anti-collagen type II antibodies, which are pathogenic in RA. Interestingly, the sympathetic neurotransmitter norepinephrine can inhibit the pro-inflammatory effects of IL-7R+ B cells, suggesting a potential regulatory mechanism that could be targeted therapeutically (65). Moreover, IL-7 has been identified as a potent chemoattractant for monocytes, which express IL-7R. The recruitment of these monocytes to the inflamed joints is mediated through the activation of the PI3K/AKT1 and ERK pathways, contributing to the chronic inflammation and joint destruction observed in RA (61). The interaction between IL-7 and IL-7R on monocytes also promotes their differentiation into osteoclasts, the cells responsible for bone resorption. This process is further enhanced by the IL-7-induced expression of RANKL on T cells, which stimulates osteoclastogenesis (66). The dual role of IL-7/IL-7R signaling in both immune activation and bone destruction highlights its potential as a therapeutic target in RA. Blocking IL-7 or IL-7R has been shown to reduce inflammation and bone erosion in animal models of arthritis. For example, treatment with anti-IL-7 antibodies in CIA mice significantly decreased monocyte recruitment, osteoclast differentiation, and joint destruction (61, 66). Similarly, soluble IL-7Rα can inhibit IL-7-induced proliferation and cytokine production by T cells, further supporting the therapeutic potential of targeting this pathway (62). In addition to its direct effects on immune cells, IL-7 also interacts with other cytokines to perpetuate the inflammatory cycle in RA. Notably, IL-7 expression is up-regulated by pro-inflammatory cytokines such as IL-6, creating a feedback loop that sustains the chronic inflammation in the synovium (67). This relationship between IL-7 and IL-6 underscores the complexity of cytokine networks in RA and the need for multi-targeted therapeutic approaches. The expanding role of IL-7 and its receptor in RA pathogenesis has led to the exploration of IL-7 blockade as a potential therapeutic strategy. Given the challenges in reconstituting T cells in RA patients, therapies that minimize the elimination of T cells while inhibiting their pathogenic functions are particularly desirable (64). The development of IL-7R-targeted therapies could offer a novel approach to managing RA by simultaneously addressing inflammation and bone destruction.

In conclusion, IL-7 and its receptor play a multifaceted role in the pathogenesis of RA, influencing T cells, B cells, and monocytes to drive inflammation and joint damage. The therapeutic targeting of the IL-7/IL-7R axis holds promise for improving disease outcomes by disrupting these pathogenic processes. Further research is needed to fully elucidate the mechanisms of IL-7/IL-7R signaling and to develop effective therapies that can modulate this pathway in RA patients.

### 3.2 Systemic lupus erythematosus

SLE is a complex autoimmune disease. The core pathological mechanism of SLE is the loss of immune tolerance to self-antigens, leading to the activation of autoreactive T cells and B cells, which produce pathogenic autoantibodies that subsequently cause tissue damage (68–70). Former studies have revealed that the soluble form of IL-7R (sIL-7R) is significantly elevated in SLE patients (71, 72). The results of Badot V et al. (71) show that sIL-7R level is significantly elevated in SLE patients, especially in patients with lupus nephritis. The serological level of sIL-7R is positively correlated with SLE disease Activity Index scores and decreases following immunosuppressive therapy. Moreover, abundant expression of IL-7R is observed in the renal biopsy specimens of SLE patients (71). Similar observation is also consistent with findings from another group (73). In this context, study (13) has confirmed that sIL-7R competes with the cell-associated IL-7 receptor, reducing excessive consumption of IL-7, thereby enhancing the biological activity of IL-7 when its amount is limited. Polymorphic analysis provided further validation for the role of IL-7 and its receptor in SLE, revealing that IL-7R single nucleotide polymorphism rs6897932 (C/T) is associated with susceptibility to SLE (74). In addition, the levels of IL-7 in tear samples of SLE patients are significantly higher than those in control group (75). Bidirectional mendelian randomization results suggest the level of IL-7 ((OR = 1.401; 95% C.I. 1.010–1.943)) was associated with a heightened risk of SLE development (76). The increased IL-7 levels contribute to the expansion and survival of autoreactive T cells, thereby exacerbating the autoimmune response (10, 77, 78). Blocking the IL-7 receptor can reduce the activation of T cells and autoimmune manifestations, and it is effective even in the late stages of the disease (77). Moreover, the balance between regulatory T cells (Tregs) and effector T cells is often impaired in SLE. In SLE patients, there is a significant increase in effector memory CD8+ T cells with low expression of IL-7Rand effecmay lead to tissue damage through 2B4-mediated cytotoxicity (79). In addition, there is an imbalance of Treg and Th17 cells in SLE patients, with increased levels of Th17 cells and related cytokines (such as IL-17, IL-6, IL-21) and decreased proportion of Treg cells (80). IL-7 has the ability to stimulate the proliferation of Th17 and Th1 cells in some autoimmune disease. And it can prompt IL-7R has the ability to stimuland Th17-associated cytokines, such as IFN-γ and IL-17 (81, 82). Therefore, these results remind us that IL-7 and its receptor may participate in the pathogenesis of SLE. Furthermore, the role of IL-7 in SLE may be closely linked to the function of Toll-like receptors (TLRs), particularly TLR7 and TLR9. TLR7 and TLR9 are endosomal receptors that recognize nucleic acids and respond to RNA and DNA, respectively, which express mainly on B cells and plasmacytoid dendritic cells (pDCs) (83, 84). In SLE patients, abnormal activation and proliferation of B cells is mediated by a variety of receptors, including TLR7 and TLR9 (85). TLR7 signaling in B cells is considered to be a key factor in the pathophysiology of SLE (86). Study (87) has also shown that TLR7 exacerbates disease progression in SLE by promoting the production of interferon-α (IFN-α) by pDCs. Conversely, loss of TLR9 leads to enhanced TLR7 signaling, which accelerates the progression of SLE (88). In TLR9-deficient mouse models, over-activation of TLR7 was strongly associated with increased autoantibodies and exacerbation of lupus nephritis (88, 89). Researches have reported that inhibiting TLR7 signaling or simultaneously activating TLR9 may help reduce the symptoms of SLE (83, 89). It has been demonstrated that the IL-7/IL-7R signaling axis is crucial for various stages of B cell development, from early progenitors to mature B cells (24). And the combination of IL-7 and TLR7 can significantly enhance the proliferation and activation of B cells and T cells. Bikker A et al. (90) have found that IL-7 alone induce T cell activation, when combined with TLR7 agonists such as Gardiquimod, is able to synergistically increase the number of proliferating B and T cells. This synergistic effect is even more pronounced in the presence of monocytes and macrophages. These studies suggest that the IL-7 and its receptor may play a role in SLE by modulating the growth and function of immune cells that produce TLR7 and TLR9.

In conclusion, IL-7 and its receptors may play a pivotal role in the pathogenesis of SLE by influencing T cell homeostasis and growth and function of B cells. The dysregulation of IL-7 signaling in SLE leads to the expansion of autoreactive T cells and the impairment of Tregs. Targeting IL-7 and its receptors represents a promising therapeutic strategy for SLE, with the potential to restore immune tolerance and reduce disease activity. Further research is needed to fully elucidate the mechanisms by which IL-7 contributes to SLE pathogenesis and to develop effective IL-7-targeted therapies.

### 3.3 Multiple sclerosis

Genetic studies have identified IL-7R as a non-HLA gene associated with MS susceptibility. Specifically, polymorphisms in the IL-7Rα gene, such as rs6897932, have been linked to an increased risk of developing MS (91–93). These polymorphisms affect the splicing of IL-7Rα, leading to variations in the production of soluble and membrane-bound isoforms of the receptor. The soluble form of IL-7Rα (sIL-7Rα) can modulate the availability and activity of IL-7, thereby influencing T cell homeostasis and immune responses (13, 94). IL-7 is essential for the survival and expansion of T helper type 17 (TH17) cells, which are implicated in the pathogenesis of MS. In experimental autoimmune encephalomyelitis (EAE), a mouse model of MS, IL-7 directly promotes the expansion of pathogenic TH17 cells, while IL-7R antagonism induces apoptosis in these cells by inhibiting the JAK-STAT5 pathway and altering the expression of pro-survival and pro-apoptotic proteins (95). This selective effect on TH17 cells, as opposed to TH1 and regulatory T (Treg) cells, underscores the potential of targeting the IL-7/IL-7R axis in therapeutic strategies for MS. Interestingly, IL-7 also promotes the differentiation of naïve T cells into T helper type 1 (TH1) cells, another subset involved in MS. High serum levels of IL-7 have been shown to predict a good clinical response to interferon-β (IFN-β) therapy in MS patients, indicating a TH1-driven disease (96). This suggests that IL-7 levels could serve as a biomarker for identifying MS subtypes and tailoring treatment strategies accordingly. The IL-7Rα gene contains response elements to IFN-β, the most commonly used immunomodulatory drug in MS. Variations in IL-7Rα haplotypes influence the receptor’s expression in response to IFN-β, which may contribute to the genetic association of IL-7Rα with MS. For instance, haplotype 2, which is protective, shows reduced splicing of exon 6 and lower production of sIL-7Rα, thereby reducing interference with receptor binding to IL-7 and TSLP (97). Moreover, the balance between membrane-bound IL-7Rα and sIL-7Rα is altered in MS patients. Lower levels of sIL-7Rα and higher membrane-bound IL-7Rα to sIL-7Rα ratios have been observed in MS patients compared to healthy controls. This skewed ratio may enhance the responsiveness of IL-7Rα+ cells, contributing to the autoimmune response in MS (94). The functional implications of IL-7 and IL-7R in MS are further supported by studies showing that sIL-7Rα potentiates IL-7 bioactivity by preventing its excessive consumption. This potentiation effect exacerbates EAE, highlighting the role of IL-7 in promoting autoimmunity (13). Additionally, IL-7 signaling in the presence of sIL-7Rα diminishes the expression of regulatory molecules such as CD95 and suppressor of cytokine signaling 1, further promoting T cell survival and proliferation (13).

In summary, IL-7 and its receptors play a multifaceted role in MS by influencing the survival, proliferation, and differentiation of T cells. Genetic variations in IL-7Rα contribute to MS susceptibility and modulate the immune response, making the IL-7/IL-7R axis a promising target for therapeutic intervention. Understanding the complex interactions between IL-7, IL-7R, and T cell subsets is crucial for developing personalized treatment strategies for MS patients.

### 3.4 Type 1 diabetes

Genetic variations in the IL-7R gene have been associated with susceptibility to T1D, suggesting a significant role in the disease’s development and progression (98–100). IL-7R blockade has shown promise in reversing established T1D in non-obese diabetic (NOD) mice. Studies have demonstrated that IL-7Rα antibody therapy can induce durable remission in newly onset diabetic mice, primarily by modulating effector T-cell function. Specifically, IL-7 increases the production of IFN-γ by CD4+ and CD8+ T cells, which are crucial in the autoimmune attack on pancreatic β-cells. Conversely, IL-7Rα antibody therapy reduces these IFN-γ-producing T cells and enhances the expression of the inhibitory receptor Programmed Death 1 (PD-1) on effector T cells, promoting immune tolerance (98, 101, 102). The soluble form of IL-7Rα (sCD127) also plays a role in T1D. Elevated levels of sCD127 have been observed in patients at the onset of T1D, influenced by islet autoantibody status and specific genetic polymorphisms within the IL-7RA gene. sCD127 can bind IL-7 and act as an antagonist to IL-7 signaling, thereby regulating T-cell proliferation. However, in the presence of high glucose levels, sCD127 becomes glycated and loses its antagonistic function, potentially compromising the regulation of IL-7-mediated T-cell survival and expansion in T1D patients (99). Further research has highlighted the unique effects of IL-7 on monocyte phenotype and function in T1D. IL-7R expression on monocytes is up-regulated in response to IL-7, and this effect is more pronounced in nonclassical monocytes. In T1D patients, IL-7R expression on monocytes is lower compared to healthy controls, indicating a potential impairment in IL-7-mediated monocyte functions. This differential expression and response to IL-7 may contribute to the pathogenesis of T1D by affecting monocyte activation and maturation (103). Regulatory T cells (Tregs) are another critical component in the context of IL-7 and T1D. IL-7 uniquely maintains FoxP3+ adaptive Tregs (aTregs), which can reverse diabetes in NOD mice. These aTregs express high levels of IL-7Rα and depend on IL-7 for their persistence. Unlike natural Tregs (nTregs), aTregs do not rely on IL-2, which is defective in T1D patients. This distinct regulation suggests that aTregs could be a viable therapeutic candidate for controlling the autoimmune response in T1D. The localization of aTregs to the pancreas, mediated by integrin-α7, is crucial for their function in controlling diabetogenic effector T cells (104, 105). Clinical trials with humanized anti-IL-7R monoclonal antibodies, such as RN168 and PF-06342674, have shown promising results in T1D patients. These antibodies effectively block IL-7Rα, leading to a significant decline in effector and central memory T cells while preserving naive T cells and Tregs. This selective inhibition of memory T cells, coupled with the down-regulation of genes associated with T cell activation and survival, suggests that IL-7R blockade can modulate the immune response in T1D. The trials also indicated that IL-7R blockade could maintain near-maximal receptor occupancy, providing a sustained therapeutic effect (102, 106).

In conclusion, IL-7 and its receptor play a multifaceted role in the pathogenesis of T1D. The modulation of IL-7 signaling through IL-7R blockade offers a promising therapeutic approach by promoting immune tolerance, altering the balance of effector and regulatory T cells, and potentially reversing established diabetes. Further research and clinical trials are necessary to fully understand the mechanisms and optimize the therapeutic strategies targeting the IL-7/IL-7R pathway in T1D.

## 4 Conclusions

IL-7 and its receptor play a crucial role in the pathogenesis of autoimmune diseases. IL-7 is essential for T cell homeostasis and survival, and its dysregulation has been implicated in various autoimmune conditions. Genetic polymorphisms in IL-7R, such as rs6897932, are associated with increased susceptibility to autoimmune diseases by affecting the expression of surface and soluble IL-7R in monocytes and T cells. IL-7R signaling is vital for the survival and expansion of pathogenic T helper type 17 (TH17) cells, which are key players in autoimmune inflammation, as seen in multiple sclerosis. Blocking IL-7/IL-7R signaling has shown therapeutic potential in reducing autoimmune responses by promoting apoptosis of effector T cells and enhancing regulatory mechanisms. Additionally, IL-7R antagonism has been effective in reversing autoimmune diabetes by inhibiting effector/memory T cells and promoting tolerance. These findings highlight the significant role of IL-7/IL-7R in autoimmune diseases and suggest that targeting this pathway could be a promising therapeutic strategy.
